# Field and lab experimental demonstration of nonlinear impairment compensation using neural networks

**DOI:** 10.1038/s41467-019-10911-9

**Published:** 2019-07-10

**Authors:** Shaoliang Zhang, Fatih Yaman, Kohei Nakamura, Takanori Inoue, Valey Kamalov, Ljupcho Jovanovski, Vijay Vusirikala, Eduardo Mateo, Yoshihisa Inada, Ting Wang

**Affiliations:** 1 NEC Laboratories America, Inc, Princeton, NJ 08540 USA; 2 Submarine Network Division, NEC Corporation, 108-8001 Tokyo, Japan; 3 Google, Inc, Mountain View, CA 94043 USA

**Keywords:** Electrical and electronic engineering, Fibre optics and optical communications

## Abstract

Fiber nonlinearity is one of the major limitations to the achievable capacity in long distance fiber optic transmission systems. Nonlinear impairments are determined by the signal pattern and the transmission system parameters. Deterministic algorithms based on approximating the nonlinear Schrodinger equation through digital back propagation, or a single step approach based on perturbation methods have been demonstrated, however, their implementation demands excessive signal processing resources, and accurate knowledge of the transmission system. A completely different approach uses machine learning algorithms to learn from the received data itself to figure out the nonlinear impairment. In this work, a single-step, system agnostic nonlinearity compensation algorithm based on a neural network is proposed to pre-distort symbols at transmitter side to demonstrate ~0.6 dB Q improvement after 2800 km standard single-mode fiber transmission using 32 Gbaud signal. Without prior knowledge of the transmission system, the neural network tensor weights are constructed from training data thanks to the intra-channel cross-phase modulation and intra-channel four-wave mixing triplets used as input features.

## Abstract (web summary)

Long-distance fiber communications still face many fundamental challenges in capacity due to nonlinearities. The authors develop a neural-network based tool to compensate nonlinearities, without prior knowledge of the transmission link, with low complexity.

## Introduction

Capacity of optical transmission systems are bound by fundamental limits in both linear and nonlinear regime^1^. Recent experiments demonstrated capacities approaching the Shannon limit in the linear regime^2–4^. This leaves the Kerr nonlinearity as one of the major limitations to increasing capacity per fiber. Nonlinear compensation (NLC) algorithms were introduced in digital coherent receivers to compensate for the pattern-dependent and deterministic Kerr nonlinearity^5,6^. These digital signal processing (DSP) NLC algorithms were based on solving the nonlinear Schrödinger equation (NLSE) which governs the propagation of optical field in the fiber^7^ in multiple steps such as digital back propagation (DBP)^5,6^. A variety of techniques were developed to reduce the complexity of NLC for a given amount of improvement, some of which reduced the required complexity by orders of magnitude such as the filtered DBP^8^. Compared to full-step DBP, at least 0.2 steps per span (SpS) and 0.6 SpS are found to be sufficient for QPSK^8^ and 16QAM^9^, respectively. The first-order linear perturbation of NLSE has led to the single-step perturbation-based pre/post-distortion (PPD) algorithm for Gaussian^10^ and root-raised cosine (RRC) pulses^11–13^. PPD was demonstrated over transoceanic distances to achieve only slightly less NLC gain than filtered DBP at 0.5 SpS^12^. Furthermore, after the initial success of filtered DBP and PPD in reducing the complexity, further reduction of complexity proved difficult to achieve. The common thread among these algorithms was that they were based on equalizing the nonlinearity based on a deterministic model of the impairment.

Recently, a different approach was taken using machine learning algorithms^14–17^. These algorithms aim to equalize nonlinear impairments directly by learning from data, rather than through emulating NLSE. These attempts remained limited in success in particular for dispersion-uncompensated links, or in scope until it was demonstrated recently through a field trial on a live-traffic cable that a simple neural network (NN) can provide NLC if it was supplemented with nonlinear impairment features^18^. A second approach^19^ showed through simulations that, NN can also be used to obtain NLC by treating the DBP steps as NN layers. However, by design, this approach requires operating on at least two samples per symbol, which induces additional complexity.

In this paper it is shown that the NN architecture demonstrated in ref. ^18^ can be simplified further so that it can achieve NLC gain at a complexity lower than filtered DBP algorithms that are based on solving NLSE, especially at the most critical regime where the available DSP resources become scarce. Furthermore, NN not only learns from the received data and generates a black-box model of the transmission, it also can guide us how to reduce the complexity through the weights by distinguishing the terms that contribute significantly from the ones that do not. Another advantage of the proposed algorithm is that since it relies only on received data to emulate the transmission model, it works without prior knowledge of the link parameters. Since the algorithm becomes free from specifics of the link design, it can be applied universally to all fiber optical communication links whether they are short-haul, long-haul, terrestrial, submarine, or whether they are legacy systems or the state-of the art. It is also shown that the algorithm is versatile and robust enough that while the training can be performed at the receiver side which is the most practical case, the equalization can be performed on the transmitter side. NLC at the transmitter has the benefit of achieving slightly better Q improvement, but more importantly the possibility of a further reduction of complexity by calculating nonlinearity features with look up tables (LUT) rather than real multiplications. Performance of the proposed universal NN-NLC algorithm is demonstrated in both lab experiment over 2800 km standard single-mode fiber (SSMF) loop and field trial over 11,017 km straight-line FASTER cable. Compared with single-step filtered DBP algorithm, NN-NLC algorithm is capable of achieving ~0.35 dB Q-factor improvement in the 2800 km SSMF transmission and attaining ~0.08 b/s/Hz higher generalized mutual information (GMI) after 11,017 km submarine distance. The results show that NN-NLC has more potential to outperform filtered-DBP especially in the regime where the degree of computational complexity is limited.

## Results

### Input features

Even though the NN algorithm needs only data to achieve a working model of the nonlinear impairment, it was found that providing the NN with nonlinear impairment features was necessary^18^. These features are provided to the NN by first calculating the intra-channel cross-phase modulation (IXPM) and intra-channel four-wave mixing (IFWM) triplets from the received symbols^10,20^. The triplets originated from the first-order perturbation of the NLSE that describes the evolution of the optical field as follows^7^:

1 $${\frac{{\partial u_{x/y}(t,z)}}{{\partial z}} + i\frac{{\beta _2}}{2}\frac{{\partial ^2u_{x/y}(t,z)}}{{\partial t^2}} = i\frac{8}{9}\gamma \left[ {\left| {u_{x/y}(t,z)} \right|^2 + \left| {u_{y/x}(t,z)} \right|^2} \right]u_{x/y}(t,z),}$$

where *u*_*x*/*y*_(*t*, *z*) is the optical field of *x* and *y* polarization, respectively, *β*_2_ is the group velocity dispersion, and *γ* is the nonlinear coefficient. In the first-order perturbation theory, the solution to Eq. (1) consists of both linear *u*_0,*x*/*y*_(*t*,*z*) and nonlinear perturbation Δ*u*_*x*/*y*_(*t*, *z*) terms^10,21^. Assuming much larger accumulated dispersion than symbol duration, the nonlinear perturbation terms for the symbol at *t* = 0 can be approximated as^22^

2 $$\Delta u_{x/y}(0,z) = \mathop {\sum}\limits_{m,n} {P_0^{3/2}} \left( {H_nH_{m + n}^ \ast H_m} \right.\left. { + V_nV_{m + n}^ \ast H_m} \right)C_{m,n},$$

where *P*_0_, *H*_*m*_ and *V*_*m*_, and *C*_*m*,*n*_ are, respectively, the launch power, symbol sequences for the *x*- and *y*-polarization, and nonlinear perturbation coefficients, *m* and *n* are symbol indices with respect to the symbol of interest *H*_0_ and *V*_0_. The triplet is defined as $T \equiv H_nH_{m + n}^ \ast H_m + V_nV_{m + n}^ \ast H_m$ in this paper. The nonlinear perturbation coefficients *C*_*m*,*n*_ can be analytically computed given the link parameters and signal pulse duration/shaping factors^10^, whereas the triplets do not depend on the link and can be calculated directly from the received symbols.

The proposed NN-NLC algorithm is divided into two stages: training and execution stages. In the training stage, the NN learns from the training data and generates a black-box model of the transmission link. In the execution stage, the nonlinear impairment is calculated based on the model, and the impairment is removed from the data.

### Training stage

**Fig. 1:**
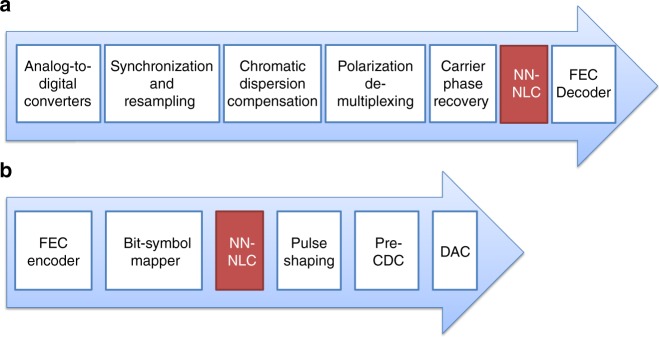
DSP flowchart with NN-NLC. **a** Receiver side, and **b** transmitter side

**Fig. 2:**
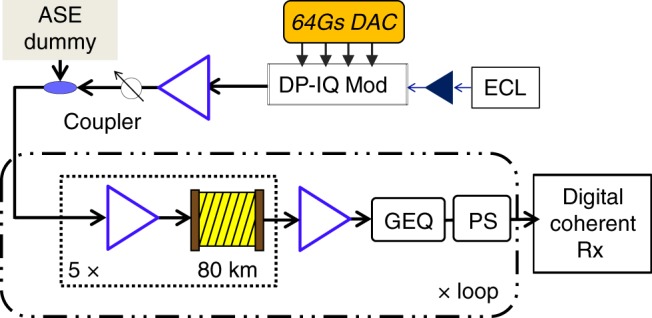
System setup of the transmitter and transmission loop. DAC: digital-to-analog converter, ECL: external cavity laser, GEQ: gain equalizer, PS: polarization scrambler

During the training stage it is necessary to have sufficient nonlinearity therefore the launch power *P*_0_ should be close to, or larger than the optimum channel power. One drawback of data-driven modeling is that the received data is corrupted not only by nonlinear impairments but also by amplified spontaneous emission (ASE) noise. This can be easily taken care of by transmitting the same training data repeatedly and averaging out the pattern-independent noise such as ASE, and inter-channel nonlinearities, while the intra-channel nonlinear impairments can be retained. NN-NLC operates on the soft data output from carrier phase recovery block shown in DSP flowchart of Fig. 1a. At the training stage NN-NLC needs to be implemented at the receiver side whereas the execution stage can be implemented at either the transmitter or receiver side, see Fig. 1. As the training does not have to operate at the data rate unlike the execution stage, and the amount of training data is not excessive, it can be performed offline to save computation cost. To test the NN-NLC algorithm with experimental data, single-channel 32 Gbaud dual-polarization (DP)-16QAM with RRC 0.01 pulse shaping as shown in Fig. 2 is generated using 64 Gsa/s DAC and transmitted over a recirculating loop testbed consisting of five spans of 80 km SSMF with 0.2 dB/km loss and 17 ps/nm/km dispersion. A digital coherent receiver running at 50-GSa/s with analog bandwidth of 20 GHz downsamples the optical waveforms for offline DSP outlined in Fig. 1a to recover the transmitted symbols. In addition, 50% chromatic dispersion compensation (CDC) is applied at the transmitter to reduce the interaction length between symbols^13^. Three uncorrelated datasets each with ~115 k symbols are generated for training, cross-validation (CV) and testing. The data pattern used in the training, CV and test datasets is measured to have maximum 0.6% normalized cross-correlation to ensure data independence.

**Fig. 3:**
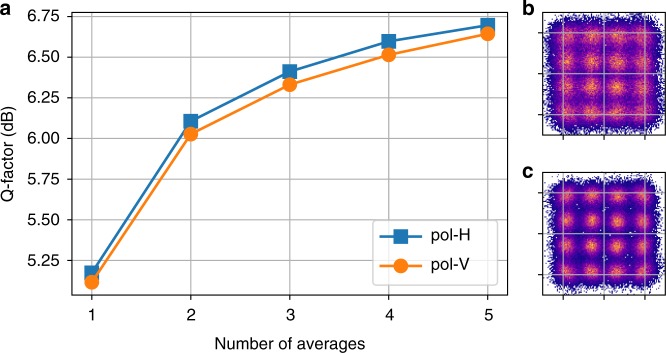
Denoising. **a** The impact of de-noising by  averaging over the training datasets on the Q-factor at SNR = 18.4 dB after 2800 km. **b** Received constellation before de-noising, and **c** after de-noising

As the training pattern is fixed at the transmitter side and the ASE noise is an independent additive Gaussian noise, the recovered symbols can be easily synchronized using a framer in practice to align the symbols in the same order such that the ASE noise can be reduced after averaging, while keeping the nonlinear interaction intact. Multiple waveform acquisition is processed, and the recovered soft symbols after carrier phase recovery are aligned to average out the additive noise. Figure 3 plots the impact of the number of acquired waveforms on the Q-factor and constellation of the training dataset received at ~2 dB higher channel power than the optimum after 2800 km transmission. About 1.6 dB Q-factor improvement is observed after averaging over just 5 acquired waveforms. The saturation curves show that the resulting cleaner constellation in Fig. 3c is able to more accurately represent the nonlinear noise than the one in Fig. 3b.

### Parameter optimization of ML model

**Fig. 4:**
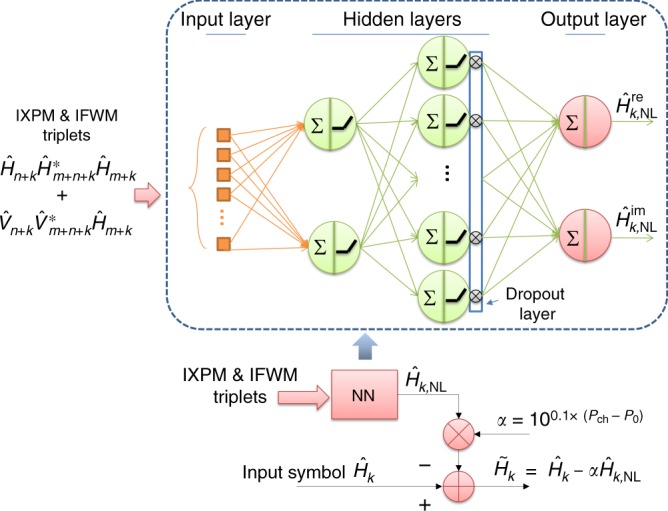
The block diagram of the proposed NN-NLC. Illustrated for pol-x only. The diagram in the dashed box describes the optimized NN architecture with two hidden layers used in the paper

There are various ML models from simple linear regression to sophisticated deep-learning models used for solving a variety of problems. Fully connected neuron network is selected to demonstrate the effectiveness of NN for NLC at a similar or even lower complexity than existing DSP algorithm. The optimized feed-forward NN model shown in Fig. 4 is constructed from an input layer with 2*N*_*t*_ triplets nodes, 2 hidden layers consisting of 2 and 10 nodes, respectively, and two output nodes corresponding to the real and imaginary parts of the estimated nonlinearity. The optimization of the number of hidden layers and the number of nodes in each layer is carried out to simplify the complexity of the NN architecture without degrading the BER performance of the derived models on the CV datasets. Note that the triplets are separated into real and imaginary parts before being fed into the NN model. Although Eq. (2) describes linear relationships among these IFWM/IXPM triplets due to the first-order perturbation, nonlinear activation function in the neuron nodes is found in our study to achieve better performance than linear function. The impact of activation function is explained in the next section. A dropout layer with probability of 0.5 is placed after the 2nd hidden layer during training only to avoid overfitting. Applying Adam learning algorithm^23^ with a learning rate of 0.001 and batch size of *B* = 100, the network is trained by transmitting known but randomly generated patterns, and searching for the best node tensor parameters that minimize the mean square error (MSE) between the transmitted and received symbols after NN-NLC, i.e.,

3 $${\mathrm{MSE}} = \frac{1}{B}\mathop {\sum}\limits_{i = 1}^B {\left| {H_i - \left( {\hat H_i - \hat H_{i,{\mathrm{NL}}}} \right)} \right|} ,$$

where $\hat H_i$ and $\hat H_{i,{\mathrm{NL}}}$, respectively, are the received symbols and estimated nonlinearity for pol-x. Although the model is trained for x-polarization data, same weights can be used to obtain a similar performance improvement for the y-polarization data too. Note that the training can be done at much slower pace than data rate to allow deep-learning algorithm to locate the appropriate NN models and compute the optimum tensor weights prior to the execution stage.

### The impact of activation function

**Fig. 5:**
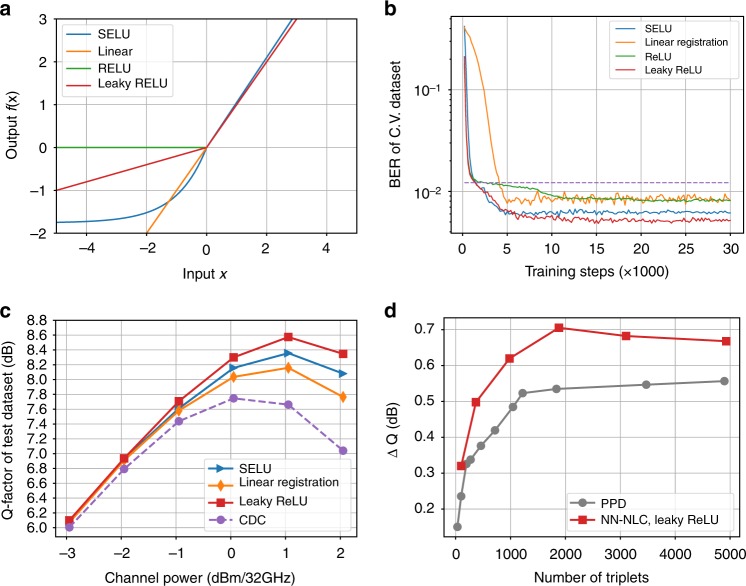
The impact of activation function on the NN-NLC algorithm. **a** Plots of different activation functions. **b** The BER optimization trace of DNN over the CV dataset in the training with different activation functions. **c** The Q-factor of DNN with different activation functions as a function of channel power. **d** The Q-factor improvement of PPD and NN-NLC with Leaky ReLU w.r.t. the optimum CDC Q

A single-channel 32Gbaud DP-16QAM is simulated over 40 × 80 km SSMF with 50% pre-CDC to compare the performance of four different activation functions plotted in Fig. 5a, namely SELU, ReLU, Leaky ReLU, and linear, on both CV and test datasets with *N*_*t*_ = 2065 triplets. Note that linear function represents just the linear regression. As shown in Fig. 5b, the training process converges faster when applying nonlinear activation function as they help alleviating the vanishing gradients problems^23^. In addition, it is found that the Leaky ReLU activation function is the optimum among these four varieties. As shown in Fig. 5b, the linear combination of these triplets shown in the linear regression curve is not as good as the cases with nonlinear activation using SELU and Leaky ReLU. It may be caused by the nonlinear interaction between these triplets in these nonlinear activation functions to account for even higher-order nonlinearities, such as 6th-order interaction between symbols. These observations are verified in simulation shown in Fig. 5c to further demonstrate the advantage using Leaky ReLU activation function as the gap between the linear regression and RELU grows at higher powers. Based on the study results, Leaky ReLU is used in our experiments to maximize the NLC gain.

To exclude the impact of the uncertainties of the experimental setup on the performance, simulation results are used to compare the NLC performance between PPD and NN-NLC algorithm as a function of the number of triplets. The calculation of perturbation coefficients are based on ref. ^13^. The Q improvement over the CDC is plotted in Fig. 5d as a function of the number of triplets at the optimum channel power of 1 dBm. The NN-NLC with Leaky-ReLU outperforms PPD by ~0.15 dB at ~2000 triplets thanks to the nonlinear activation function in the neuron nodes.

### Triplets selection

After cleaning up the ASE noises in the received training dataset, IXPM & IFWM triplets are calculated according to Eq. (2). In systems with large dispersion, symbols can overlap and interact with thousands of neighboring symbols. In order to keep the complexity low, the number of triplets should be kept low by only including the ones that contribute the most. This requires establishing a selection criterion^10,13^. In previous work^18^, nonlinear perturbation coefficients *C*_*m*,*n*_ were used as a way to estimate which triplets contributed the most. Only the triplets that satisfied the criterion *C*_*m*,*n*_ > *κ* was retained where *κ* was a free parameter to adjust the trade off between complexity and performance. Even though *C*_*m*,*n*_ were not used in the execution stage, their computation during the training stage still required accurate link and transmission system parameters^18^. Considering that the *C*_*m*,*n*_ has a hyperbolic dependence on *m* and *n*, we propose to choose all the triplets with index pairs *m*, and *n* that satisfies the following criterion that is independent of link parameters

4 $$|n| \le \min \left\{ {\frac{{\rho \left\lceil {L/2} \right\rceil }}{{|m|}},\left\lceil {\frac{L}{2}} \right\rceil } \right\},$$

where min{⋅} takes the minimum of its arguments, $\left\lceil \cdot \right\rceil$ stands for the ceiling function, *L* determines the largest value of *m*, and *n*, and *ρ* controls the maximum of the *m*, *n* product. The values required for *L* and *ρ* are not too restrictive as long as a sufficiently large number of triplets are chosen to initialize the training. Through iterative trimming during the training stage, as discussed below, the excess triplets can be removed before the execution stage.

### Execution stage

During the training stage, the performance of the model is checked against the CV dataset only to optimize the NN model parameters. Afterwards the learned model is applied to the uncorrelated test dataset for all channel powers in the execution stage. The block diagram of the proposed NN-NLC is shown in Fig. 4. Given the symbol of interest $\hat H_0$ centered at the middle of pattern length *L*, the IXPM and IFWM terms are calculated and fed into the NN model described in the dashed box of Fig. 4 to estimate the nonlinearity. The estimated $\hat H_{0,{\mathrm{NL}}}$ is then scaled by the channel power (*P*_ch_) of the test dataset with respect to the reference channel power (*P*_ref_) of the training data used for deriving the model, i.e.,

5 $$\alpha = 10^{0.1 \times (P_{{\mathrm{ch}}} - P_{{\mathrm{ref}}})}.$$

The estimated $\hat H_{0,{\mathrm{NL}}}$ is subtracted from the original symbol of interest before being sent to next DSP block, for instance, the FEC decoding in Fig. 1a.

### Complexity

Since the complexity of real multiplications could be four times as much as addition operation^24^, only real multiplication is taken into account when comparing the complexity of the NLC algorithm. The NN model shown in the dashed box of Fig. 4 requires 2*N*_*t*_ × 2 + 2 × 10 + 10 × 2 = 4*N*_*t*_ + 40 real multiplications because of three cross-layer tensor interaction. Note that the activation function Leaky ReLU() in the hidden nodes and IXPM/IFWM triplets computation are assumed to be implemented in LUT. After scaling the estimated nonlinearity term, the number of real multiplication per symbol for the proposed NN-NLC shown in Fig. 4 can be summarized as

6 $$4N_t + 42.$$

Therefore, reducing the number of triplets *N*_*t*_ is the most effective way to lower the complexity of the NN-NLC algorithm in our model.

**Fig. 6:**
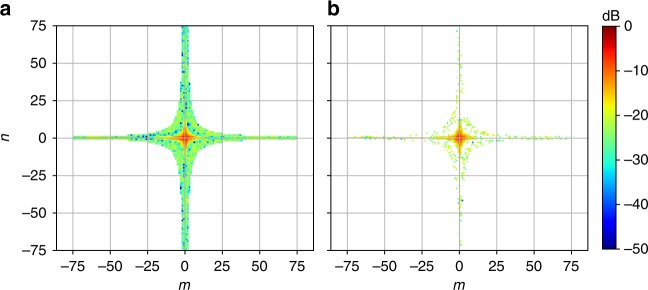
Density plots. The density plot of the input layer weights of the NN model at **a** initial *N*_t_ = 1929 and **b** *N*_t_ = 615 triplets after iterative trimming (*κ* = −22 dB). Colorbar shows the magnitude of the weights

**Fig. 7:**
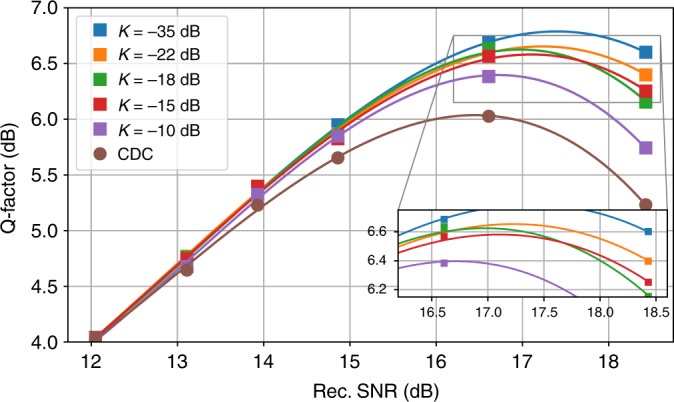
Trimming threshold. The impact of trimming threshold *κ* on the NN-NLC with Leaky ReLU at the receiver side after 2800 km transmission

**Fig. 8:**
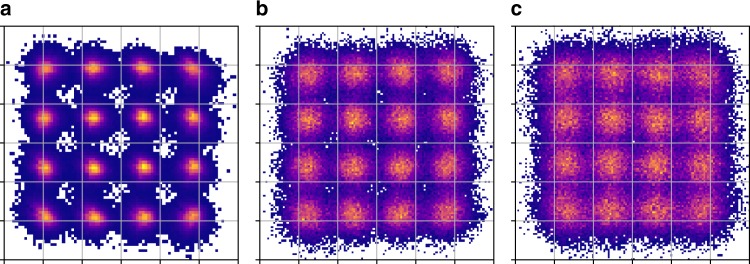
Constellations. **a** Pre-distorted symbols at transmitter side; recovered constellation at the receiver **b** with and **c** without Tx-side NN-NLC. Received SNR = 18.4 dB after 2800 km transmission

As shown in Fig. 6a, with the initial *N*_*t*_ = 1929 triplets, some of the input tensor weights *W*_*m*,*n*_ in the trained model show much smaller contribution to the signal nonlinearity than the center ones. As a result, the number of triplets *N*_*t*_ can be further reduced by only keeping those weights larger than a specified threshold *κ*, i.e., *W*_*m*,*n*_ > *κ*. After trimming off the weights *W*_*m*,*n*_ that are less than *κ* = −22 dB, the remaining 615 triplets are re-trained in the NN model and the new density plot of the input tensor weights *W*_*m*,*n*_ is shown in Fig. 6b. Figure 7 plots the impact of the trimming threshold *κ* on the performance improvement of the NN-NLC as a function of received SNR after 2800 km transmission. At the optimum received SNR, the NN-NLC algorithm at trimming threshold *κ* < −15 dB achieves >0.5 dB Q improvement over CDC. Larger Q improvement at the highest received SNR further confirms the NN model is able to accurately predict the signal nonlinearity. At the optimum received SNR, Q value improvement is adjusted from 0.2 to 0.4 dB by varying the complexity through adjusting *κ* from −35 to −15 dB, as shown in Fig. 7. Training the NN is more practical at the receiver side, however, computation of triplets at the receiver side requires the use of soft symbols. To reduce complexity further, it is better to execute the NN-NLC block at the transmitter side and use a LUT to store the triplet values rather than calculate them online. The LUT size could scale as large as *M*^3^ where *M* is the constellation size. Figure 1b shows the DSP block diagram of the NN-NLC at the transmitter side. Figure 8a shows the constellation generated after applying pre-distortion calculated by the NN at the transmitter side with *κ* set to −22 dB.

**Fig. 9:**
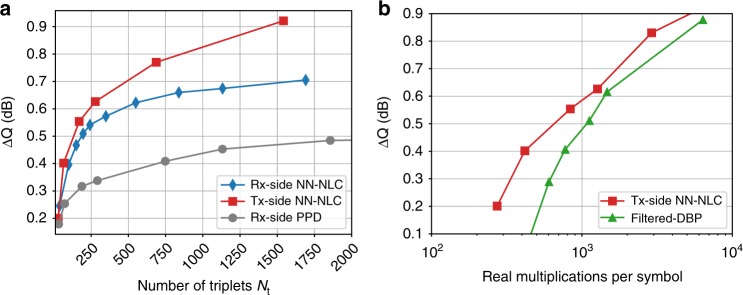
The measured performance of the NN-NLC algorithm at 2800 km. **a** The comparison of Q-factor improvement between Rx/Tx-side NN-NLC and Rx-side PPD. **b** The comparison of Q-factor improvement between Tx-side NN-NLC and filtered-DBP versus the number of multiplications

Performance of the transmitter side compensation is compared with the receiver side compensation in Fig. 9a. Even though the NN is trained at the receiver side, the transmitter side compensation performs better in all the ranges but especially at the high complexity end. This improvement is expected considering that at the transmitter side the NN model works on the clean transmitted symbols. Moreover, once the nonlinearity is mitigated at the transmitter, the receiver DSP algorithm works on the signals with reduced nonlinearity and cycle slip rate^25^. Tx-side NN-NLC outperforms PPD by more than 0.4 dB, which is attributed to the learning features and nonlinear Leaky ReLU() activation functions. Compared to the recovered constellation without NN-NLC algorithm shown in Fig. 8c, the transmitter-side NN-NLC can significantly improve the constellation quality as plotted in Fig. 8b.

**Fig. 10:**
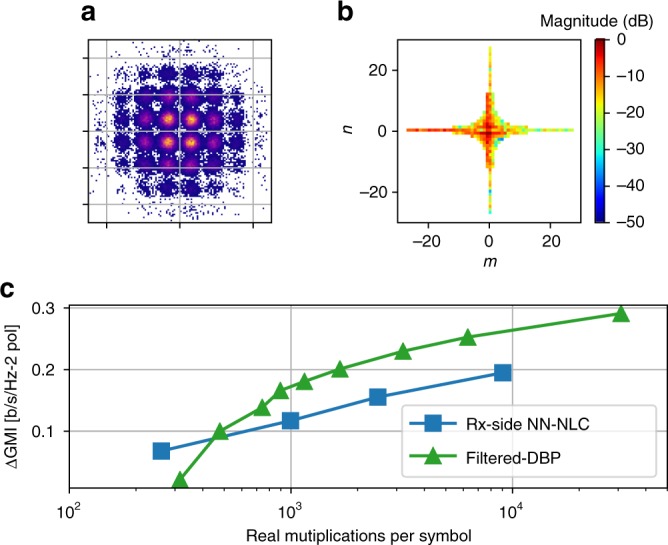
Undersea cable performance. **a** The received PS-64QAM constellation after de-noising; **b** the density map of the input layer tensors; **c** GMI performance of Rx-side NN-NLC and filtered-DBP

One of the most important criteria for a practical NLC algorithm is its low complexity while providing a significant Q improvement. Complexity of the NN-NLC was compared with NLC algorithms based on perturbation approach whose complexity also scales with the number of triplets^18^. To establish the performance of the NN-NLC with respect to existing NLC algorithms in terms of the performance-complexity trade off, the comparison is extended to the filtered-DBP technique. Filtered-DBP is chosen for comparison because it is a well-established, low-complexity^8,9^ technique where the complexity versus improvement trade-off can be easily adjusted and it provides gain as long as it has sufficient number of steps. In filtered-DBP, the number of steps can be reduced by filtering the intensity waveforms by a low-pass filter (LPF) at the nonlinear phase rotation stage. The optimal bandwidth for the Gaussian-shaped LPF is found to be 5, 1, 1, and 0.5 GHz for 1, 5, 7, and 35 spans per step (SpS). The optimum scaling factor *ξ* used to de-rotate the signal’s phase is ~0.7 for all cases. The Q performance improvement over CDC is plotted in Fig. 9b to show that Tx-side NN-NLC is capable of matching the performance of the filtered-DBP even at higher complexity than 2000 real multiplications per symbol while still keeping the performance advantage at lower complexity over filtered-DBP. More importantly, NN-NLC performs significantly better at the lowest complexity end. The complexity of filtered-DBP is calculated based on Eq. (9) in ref. ^9^ by assuming FFT size of 4086. The performance of NN-NLC is further tested on an 11,017 km commercial FASTER submarine cable together with live traffic. Digital subcarrier modulation (DSM) 4 × 12.25Gbaud capacity-approaching probabilistic-shaped (PS) 64QAM^26,27^ at RRC 0.01 with 50 MHz guard band carrying in total 300 Gb/s bit rate is used as the probe signal in 50 GHz WDM configuration. The details of the system setup and optical spectra can be found in^18^. After applying de-noising through averaging over ASE approach, the received PS-64QAM constellation at 2 dB channel pre-emphasis is shown in Fig. 10a. Note that generalized mutual information (GMI)^28^ is used for accurately measuring the gain of NN-NLC for PS-64QAM format. Figure 10c compares the performance of NN-NLC and filtered-DBP with respect to the CDC only as a function of computation complexity. Note that the received PS-64QAM soft symbols are first hard-decoded into 64 symbols in order to avoid the multiplication involved in computing triplets. Once again it is found that NN-NLC performs better than filtered-DBP when the complexity is less than ~500 real multiplications per symbol. It is expected that Tx-side NN-NLC is likely to further improve the performance gain. Figure 10b plots the density map of the input-layer nodes weights after training with 240 triplets.

Even though NN-NLC lowers the complexity in terms of required number of multipliers, a comparison of this algorithm with existing algorithms in term of detailed circuit design is not studied. Nevertheless, being a feed-forward algorithm, NN-NLC is highly parallelizable as it would be required for high-speed transponders. The proposed NN-NLC is experimentally demonstrated in both lab testbed and field cables to show the system-agnostic performance without prior knowledge of the transmission link parameters such as dispersion, fiber nonlinearity, and fiber length.

## Data Availability

The datasets generated during the current study are not publicly available due to restrictions from commercial privilege, but portions of the data are available from the corresponding author on reasonable request.

## Code availability

Example code is included for calculating the triplets, and performing the training and testing stages at http://www.nec-labs.com/~ons/NICNN/
